# Optimization of factors affecting the rooting of pine wilt disease resistant Masson pine (Pinus massoniana) stem cuttings

**DOI:** 10.1371/journal.pone.0251937

**Published:** 2021-09-10

**Authors:** Ting Pan, Xue-lian Chen, Yan-ping Hao, Chun-wu Jiang, Song Wang, Jin-shan Wang, Qiang Wei, Shi-juan Chen, Xiao-song Yu, Feng Cheng, Liu-yi Xu

**Affiliations:** 1 Department of Forest Tree Genetic Breeding, Anhui Forestry Academy, Anhui, China; 2 Key Laboratory of State Forestry Administration on Prevention and Control of Pine Wood Nematode Disease, Hefei, Anhui, China; 3 Washan State-own Forest Farm in Quanjiao, Chuzhou, Anhui, China; Georgia Southern University, UNITED STATES

## Abstract

Pine wilt disease (PWD) is a devastating disease affecting trees belonging to the genus *Pinus*. To control the spread of PWD in the Masson pine forest in China, PWD resistant Masson pine clones have been selected by the Anhui Academy of Forestry. However, because Masson pine is a difficult-to-root species, producing seedlings is challenging, especially from trees older than 5 years of age, which impedes the application of PWD resistant clones. In this study, we investigated the factors affecting rooting of PWD resistant clones and established a cheap, reliable, and simple method that promotes rooting. We tested the effects of three management methods, four substrates, two cutting materials, two cutting treatments, and three collection times on the rooting of cuttings obtained from 9-year-old PWD resistant clones. Rooting was observed only in stem cuttings treated with the full-light automatic spray management method. Additionally, stem cuttings showed a significantly higher rooting rate and root quality than needles cuttings. Compared with other substrates, stem cuttings planted in perlite produced the longest adventitious root and the highest total root length and lateral root number. Moreover, stem cuttings of PWD resistant clones collected in May showed a significantly higher rooting rate and root quality than those collected in June and July. Moreover, stem cuttings prepared with a horizontal cut while retaining the needles showed significantly higher rooting rate and root quality than those prepared with a diagonal cut while partly removing the needles. This study promotes the reproduction of seedlings of PWD-resistant Masson pine clones which helps control the spread of PWD, meanwhile, provides a technical reference for the propagation of mature pine trees via cuttings.

## Introduction

Masson pine (*Pinus massoniana*) is a unique and the main afforestation tree species native to China. Widely distributed within the range of 21°41’–33°56’N (latitude) and 102°10’–123°14’E (longitude), the Masson pine forest covers an area of 14.3 million hm^2^ in China. Masson pine trees have a wide range of applications, including the pine resin production, construction, paper products, and health care products. Moreover, Masson pine is a pioneer tree species that can withstand barren and arid soil, and plays a key role in the afforestation of barren mountains, conservation of water and soil, and improvement of the ecological environment [1]. However, the safety of Masson pine forest is seriously threatened by pine wilt disease(PWD) [2].

PWD is reportedly caused by pinewood nematode (PWN) infection [3], a bacterium carried by the PWN [4], or a combination of these two factors [5]. In Asia, PWN is mainly dispersed by *Monochamus alternatus*, a flying insect [6]. Once infected, the whole Masson pine tree wilts within 2–3 months [7]. Current measures used to control PWD such as cleaning dead wood, injecting nematicides, and reducing insect vectors using insecticides or natural enemies have mostly been found ineffective [8]. Although government agencies in China has made major financial investments to prevent the spread of PWD which was first discovered in China in 1982 [9], the area affected by the PWD epidemic has continued to expand, reaching 1.1146 million hm^2^ in 2019 [10]. According to incomplete statistical records released by the State Forestry Administration of China in 2013, approximately 5 million m^3^ of Masson pine trees have been killed by the PWN as of 2013. The rapid death of Masson pine trees has not only caused huge losses to the timber industry of China but also resulted in widespread ecological impacts [2, 11, 12].

It is worth noting that not all Masson pine trees die after being invaded by the PWN, i.e., some trees are resistant [13]. The cultivation and application of resistant pine trees may be the most economical and effective means of controlling the spread of PWN. This strategy was successfully adopted by Japan; consequently, the survival rate of the progeny of selected Japanese red pine and black pine increased by 18% and 35%, respectively, compared with that of the non-selected population [14]. Moreover, Hirao *et al*. [15] identified a novel PWN resistance locus in black pine. In 2000, the Anhui Forestry Academy launched a breeding project to study PWN resistance of Masson pine in Anhui, China, in cooperation with the government of Japan. An extensive collection of Masson pine germplasm resources was infected with PWN in multiple replicates, and 212 Masson pine clones with four levels of PWN resistance were identified in 2008 [16–18]. In these clones, the overall incidence of PWD was reduced by 63.7% compared with the non-selected Masson pine population [19]. Subsequent studies revealed that Masson pine clones showing high levels of PWN resistance were more effective in inhibiting PWN invasion and reproduction [20] and expressed the *oleoresin terpenoid synthase* gene to higher levels than clones exhibiting low levels of PWN resistance [21, 22].

PWD resistant clones must be propagated vegetatively since vegetative propagation ensures the preservation of the PWN resistance trait and enables the production of a large number of seedlings. Cutting is the most convenient method of vegetative propagation, as it is simple and efficient. However, Masson pine is a difficult-to-root tree species [23]. It needs to generate callus before it takes root and there are endogenous substances that inhibit its rooting, including phenols, flavonoids and abscisic acid [24]. There are reports study on Masson pine cuttings in many provinces of China including Guangdong [25], Hubei [26], Guangxi [27], Hunan [28], Fujian [29], Chongqing [30], Jiangxi [31], Jiangsu [32], Zhejiang [33], and Guizhou [34]. According to these studies, factors such as the collection season, growth substrate, cutting material, cutting treatment and hormonal treatment affect the rooting behavior of Masson pine cuttings. However, due to different climates, the growth patterns and optimal cutting treatments of Masson pine reported vary from province to province. In addition the age of the mother tree from which cuttings are collected influence the rooting rate of Masson pine. As the concentration of rooting inhibitors in the cuttings increases with the age of the donor tree, cuttings lose rooting capacity quickly as the donor tree ages, especially after the donor plant gets older than five when Masson pine gets mature [35]. In the existing reports, most donor trees used are under 6 years old. Little is known about the cutting method for mature Masson pine. In a few reports involving cuttings of mature Masson pine, the rooting rate of the cuttings is low which is not acceptable for cutting seedling production. Lin [36] reported that the average rooting rate of cuttings from five 9-year-old Masson pine clones was 31.0%. Ji et al. [35] reported that the rooting rate of cuttings collected from fifteen 8-year-old Masson pine plants was 22%. The PWN resistant clones planted in Quanjiao County, Anhui Province, China were 9-year-old when this research started. And before our study, there is no report on Masson pine cutting in Anhui and no cutting tests with the PWN resistant clones had been successful. To figure out a cutting method for 9-year-old PWN resistant clones planted in Anhui and realize its cutting propagation, we examined the effects of different cutting management methods, growth substrates, cutting materials, cutting treatments, and cutting collection time on rooting of PWN resistant clones and summarized the optimal cutting methodology.

## Materials and methods

### Plant material

Masson pine (*Pinus massoniana*) clones resistant to PWN were grafted in 2008 and preserved at the Washan State-own Forest Farm (32°10’N, 117°92’E) in Quanjiao County, Anhui Province, China. Thick, disease- and insect-free stems of these clones, with full top buds, were harvested, partially immersed in water to maintain freshness, and brought back to the Pine Wilt Disease Resistance Horsetail Test Base (31°85’N, 117°18’E) located in Hefei, Anhui Province. The stems were sliced horizontally to prepare 8-cm cuttings, and the needles were preserved. Needles bunch used for cutting test were collected from the stems brought back.

### Factors affecting the rooting of Masson pine cuttings

The effects of five different factors on rooting were tested in this study: management method, growth substrate, cutting material, cutting treatment, and cutting collection time. In each experiment, the cuttings were disinfected by soaking in carbendazim at a concentration of 500 ppm for 1 min. The disinfected cuttings were dipped into a rooting powder (provided by Xinyi Forestry Institute, Guangdong Province, China) and then planted into the substrate at a depth of 3 cm in rows and spacing of 5 cm × 5 cm row-to-row. The specific design of each experiment is described below.

### Management method

Three different management methods were tested: fully enclosed internal circulation, fully enclosed intermittent spraying, and full-light automatic spraying.

In the fully enclosed internal circulation experiment, the seedbed (2.5 m length × 1.3 m width × 0.3 m height) contained a 20-cm deep layer of stones at the bottom, followed by a 10-cm thick layer of substrate on top. The substrate was fully saturated with water before planting the stem cuttings. Immediately after planting, an arch canopy with a height of 0.5 m was set up above the seedbed using a transparent plastic film, and all sides of the canopy were sealed. A shade net, providing 75% shading, was established 1.5 m above the canopy. The experimental set up was monitored every morning to observe condensation on the film. If no condensation was observed, the film was uncovered, and the substrate was saturated again with water. Then the film was resealed. This treatment was conducted four times in October and December of 2017 and May and August of 2018.

The fully enclosed intermittent spray method was performed as described previously [37]. The seedbed (11 m × 1.3 m × 0.2 m) contained a 5-cm deep layer of stones (bottom), 5-cm thick layer of sand (middle), and 10-cm thick layer of substrate (top). The substrate was moistened with water, and its humidity level was maintained at approximately 50%. Stem cuttings were planted in the substrate and sprayed with mist to moisten the needles and raise the air humidity level above 95%. Then a canopy and shade net were established, as described above. Ventilation and mist were applied twice each day in the morning and evening to refresh the air in the canopy and maintain the humidity level of the air and substrate. This experiment was conducted four times in October and December of 2017 and May and August of 2018.

In the full-light automatic spray experiment, the seedbed (11 m × 1.3 m × 0.5 m) contained a 25 cm of stones (bottom layer), 15 cm of sand (middle layer), and 10 cm of substrate (top layer). The substrate was fully saturated with water before planting the cuttings. The cuttings were then sprayed with water using the LK-100 type micro-sprinkler irrigation intelligent controller and micro spray piping system (purchased from the Chinese Academy of Forestry, Beijing, China). The system was programmed to run for 20 s to moisten the needles when the water film on their surface decreased by 75%. This test was conducted twice in May and August of 2018.

In all three treatments described above, the seedbeds were constructed using bricks, and the substrate was composed of perlite. Each treatment was performed in six replicates.

### Cutting material

In July 2018, stem and needle cuttings were invoked as materials for cutting tests. The experiment adopted the full-light automatic spray cutting management method, with perlite as the substrate. Each treatment was repeated six times.

### Substrate type

In May 2018, four substrates were compared: subsurface soil: burnt soil: sand (4:3:3) mixture (hereafter referred to as mixed substrate), sand, perlite, and nutrient soil. Different substrates were tested using the full-light automatic spray management method. Each treatment was performed in six replicates.

### Cutting treatment

In June 2018, two stem cutting treatments were tested. In the first treatment, all the needles on the cuttings were retained, and the cuttings were trimmed using a horizontal cut (hereafter referred to as intact/horizontal-trim cuttings). In the second treatment, needles were removed from the bottom 5 cm of the cuttings, and the cuttings were trimmed using a diagonal cut (hereafter referred to as partially cleaned/diagonal-trim cuttings). Both these treatments were performed using the full-light automatic spray management method, with perlite as the substrate. Each treatment was performed in six replicates.

### Collection time

Stem cuttings were collected in May, June, and July of 2018. At each collection time, cuttings were collected, and their rooting was tested using the full-light automatic spray management method, with perlite as the substrate. Each treatment was performed in six replicates.

### Data collection and statistical analysis

For experiments carried out in 2017 and 2018, data were collected in September 2018 and March 2019, respectively. In the different management method experiments, data were collected only on the rooting rate. In all other experiments, data were collected on rooting rate, the number and diameter of adventitious roots, length of the longest adventitious root, total root length, and the number of lateral roots. The rooting effect index of each treatment was calculated using the following equation:

$$\mathrm{Rooting}\;\mathrm{effect}\;\mathrm{index}=\frac{\mathrm{Average}\;\mathrm{root}\;\mathrm{length}\times \mathrm{Average}\;\mathrm{root}\;\mathrm{number}\times \mathrm{Rooting}\;\mathrm{rate}}{\mathrm{Total}\;\mathrm{number}\;\mathrm{of}\;\mathrm{cuttings}}$$

All data were inputted into Statistical Product and Service Solutions (version 22) for analysis of variance (ANOVA).

## Results

### Management methods

Management methods had a substantial impact on the rooting rate (Table 1). Stem cuttings of resistant clones did not show rooting in the totally enclosed internal circulation and totally enclosed intermittent spray treatments. The rooting rates of stem cuttings in the full-light automatic spray treatment were 61% and 18% in May and July of 2018, respectively. These results indicated that the full-light automatic spray management method is more suitable for propagating the stem cuttings of resistant clones compared with the other two management methods.

**Table 1:** Effect of management methods on the rooting rate of PWD resistant Masson pine stem cuttings.

Management method	Rooting rate (%)^a^			
	September 2017	December 2017	May 2018	July 2018
Fully enclosed internal circulation	0	0	0	0
Fully enclosed intermittent spray	0	0	0	0
Full-light automatic spray	na	na	61	18

^a^na, not applicable.

### Cutting material

Rooting rates and other physiological aspects (average adventitious root diameter, length of the longest adventitious root, number of lateral roots, and rooting effect index) of stem cuttings of resistant clones were significantly superior than those of needle cuttings (Table 2). The total root length and lateral root number of stem cuttings were >10-fold greater than those of needles cuttings. A similar difference was observed in the rooting effect index of stem cuttings (0.52) and needle cuttings (0.00, and it was 0.0042 with more accurate representation). The rooting effect index considers three important aspects of rooting (total root length, root number, and rooting rate), which makes it an ideal comprehensive index for evaluating the rooting ability of Masson pine cuttings [38]. Overall, our results indicated that stem cuttings of resistant clones possess better rooting ability than needle cuttings, when managed with the full-light automatic spray method.

**Table 2:** Effect of substrate on the rooting rate and other root related traits of PWD resistant stem cuttings.

Substrate	Rooting rate (%)^a^	Root measurements^a^					
		No. of adv. roots	Adv. root diameter (mm)	Longest adv. root length (cm)	Total root length (cm)	No. of lateral roots	Rooting effect index
Mixed substrate	31.87c	3.33	0.90	7.58b	53.35b	32.17b	1.68b
Sand	75.17a	8.55	1.00	13.21b	122.10ab	144.91ab	5.20ab
Perlite	60.52ab	8.90	0.99	22.92a	265.65a	204.50a	9.93a
Nutrient soil	45.77bc	4.00	0.88	12.89b	90.46b	74.17ab	2.47b

^a^Means with different lowercase letters in the same column are significantly different (*p* < 0.05; Turkey’s comparison test). adv., adventitious.

### Substrates

The type of substrate used in the experiments had a significant effect on the rooting rate, length of the longest adventitious root, total root length, number of lateral roots, and rooting effect index of resistant clones stem cuttings (*p* < 0.05) (Table 3). The rooting rate of cuttings planted in sand (75.17%) was significantly higher than that of cuttings planted in nutrient soil (45.77%) and mixed substrate (31.87%), while there was no significant difference in rooting rate between cuttings planted in sand and those planted in perlite (60.52%). Cuttings planted in perlite produced the most longest adventitious root (22.92 cm) and showed the highest total root length (265.65 cm) and lateral root number (204.50). The rooting effect index was also the highest in perlite (9.93) among the four substrates and was significantly higher than the rooting effect index obtained in nutrient soil (2.74) and mixed substrate (1.68); These results suggest that perlite is the best substrate for rooting the stem cuttings of PWD resistant clones when managed with the full-light automatic spray method. The rooting effect index obtained in the sand was 5.20 which ranked second in the four substrates and showed no significant difference compared with that of perlite. Considering the rooting rate together, sand is a good alternative substrate to perlite.

**Table 3:** Effect of the cutting material on rooting rate and other root related traits of PWD resistant stem cuttings.

Cutting material	Rooting rate (%)^a^	Root measurements^a^					
		No. of adv. roots	Adv. root diameter (mm)	Longest adv. root length (cm)	Total root length (cm)	No. of lateral roots	Rooting effect index
Stem	17.98a	3.33	1.86a	12.89a	73.89	32.27a	0.52a
Needle brunch	2.68b	1.75	0.61b	1.85b	2.80	1.50b	0.00b

^a^Means with different lowercase letters in the same column are significantly different (*p* < 0.05; Turkey’s comparison test). adv., adventitious.

### Cutting treatments

The rooting rate and other root related traits including, average adventitious root diameter, longest adventitious root length, total root length, lateral root number, and rooting effect index, of partially cleaned/diagonal-trim cuttings were significantly lower (*p* < 0.05) than those of intact/horizontal-trim cuttings (Table 4). On the other hand, the adventitious root number of partially cleaned/diagonal-trim cuttings was higher than that of intact/horizontal-trim cuttings; however, the difference was not significant (Table 4). These data suggest that retaining the needles and trimming the cuttings horizontally enhance the rooting rate of PWD resistant stem cuttings when managed with the full-light automatic spray method.

**Table 4:** Effect of cutting treatments on the rooting rate and other root related traits of PWD resistant stem cuttings.

Cutting treatment	Rooting rate (%)^a^	Root measurements^a^					
		No. of adv. roots	Adv. root diameter (mm)	Longest adv. root length (cm)	Total root length (cm)	No. of lateral roots	Rooting effect index
Partially cleaned/diagonal trim	9.08b	3.25	1.04b	9.86b	34.73b	29.88b	0.31
Intact/horizontal trim	32.99a	2.14	1.99a	19.99a	131.47a	124.86a	5.16

^a^Means with different lowercase letters in the same column are significantly different (*p* < 0.05; Turkey’s comparison test). adv., adventitious.

### Collection time

Significant differences (*p* < 0.005) were observed in rooting rate and other root related traits of stem cuttings collected among the three collection times (Table 5). Cuttings collected and planted in May showed the highest rooting rate (60.52%), adventitious root number (8.90), lateral root number (204.50), and rooting effect index (9.93) and the longest adventitious root (22.92 mm) and total root length (265.65 cm) but the lowest average adventitious root diameter (0.99 mm). Cuttings collected and planted in June ranked second, while those collected and planted in July ranked third in all seven characteristics; the only exception was the adventitious root number, which was higher in July than in June. Overall, cuttings collected and planted in May showed a higher rooting potential than those collected and planted in June and July. This suggests that May is the most suitable time for the propagation of PWD resistant clones.

**Table 5:** Effect of collection time on the rooting rate and other root related traits of PWD resistant stem cuttings.

Collection time	Rooting rate (%)^a^	Root measurements^a^					
		No. of adv. roots	Adv. root diameter (mm)	Longest adv. root length (cm)	Total root length (cm)	No. of lateral roots	Rooting effect index
May	60.52a	8.90a	0.99b	22.92a	265.65a	204.50a	9.93a
June	32.99b	2.14b	1.99a	19.99ab	131.47ab	124.86ab	5.16ab
July	17.98b	3.33b	1.86a	12.89b	73.89b	32.27b	0.52b

^a^Means with different lowercase letters in the same column are significantly different (*p* < 0.05; Turkey’s comparison test). adv., adventitious.

## Discussion

In the totally enclosed internal circulation and totally enclosed intermittent spraying management methods, all cuttings planted in May and July of 2018 withered, and most cuttings planted in October and December of 2017 stayed alive but did not root. This was probably caused by the climate of Hefei. In Hefei, the average temperature in May and July of 2018 was 26°C and 34°C respectively, with the highest temperatures being 34°C and 38°C respectively. The temperature in the greenhouse was more than 10°C higher than that in the external environment, and the sunshade nets could not reduce the temperature in the greenhouse below 30°C because of high the ambient temperature and strong sunlight intensity. Excessively high temperature increases leaf transpiration, leading to wilting [39]. In September (autumn season), the temperature in Hefei dropped rapidly. In the period from November 2017 to April 2018, the minimum temperature was below 10°C, and although the temperature inside the greenhouse was higher than that outside, it did not stay above 15°C when the sunshade was removed. Low temperature inhibits metabolism in cuttings and also prevents rooting [40]. In the full-light automatic spray treatment, the cuttings planted in May and August could be maintained in an environment with mild temperature and humidity because the spray decreased the ambient temperature and increased moisture in the air. Thus, mild environmental conditions guaranteed the survival of cuttings and stimulated their rooting. Moreover, the full-light automatic spray method does not require shade, and the cuttings are able to photosynthesize under adequate light, which is conducive to the rooting of cuttings. In pea, cuttings preserved in the dark did not root, and the number of roots increased with the increase in irradiance of the aerial parts of cuttings [41]. Because the temperature in Hefei is low in October and December of 2018, the cuttings planted during these months could not take root in the open air. Moreover, the water in the micro-spraying system is likely to freeze when the temperature reaches below zero. Therefore, the full-light automatic spraying management method is not applicable for seedlings planted in October and December. To propagate PWD resistant Masson pine clones in autumn and winter in Hefei, a management method is needed that combines the full-light automatic spraying method with temperature control system.

The rooting rate of needles cuttings planted in perlite was 2.68%, which was significantly lower than that of stem cuttings. Moreover, the length and diameter of adventitious roots of needles bunch cuttings were much lower than those of stem cuttings. Adventitious root stimulation and formation depend on the level of plant hormones and nutrients in cuttings [42], and the initial endogenous pool of nutrients in cuttings plays an important role in rooting [43]. Perlite is a nutrient-free substrate. The nutrient content of needles was not sufficient for rooting when no fertilization was performed in this cutting test.

The rooting rate of cuttings was significantly lower in the mixed matrix than in sand or perlite. Similarly the rooting rate of cuttings in nutrient soil was significantly lower than that in sand. These differences in results were probably caused by differences in the matrix particle size and water permeability of the substrates. All four substrates were tested using the full-light spray method, which requires repeated spraying. The mixed substrate and nutrient soil, both of which have small particle size and poor water permeability, accumulated the sprayed water, thus decreasing the oxygen level at the base of cuttings. Poor aeration in waterlogged conditions leads to the decay of cuttings before root initiation [44]. In willow, the submergence of cuttings in flowing water decreased the rooting rate and total root mass [45]. Sand and perlite have larger particle size and good water permeability. Thus, both these substrates showed no accumulation of water, which most likely improved oxygen flow. A well-aerated environment increases respiration at the base of cuttings [46], thus promoting rooting. In November 2016 we conducted a preliminary cutting experiment with the totally enclosed intermittent spraying management methods in the artificial climate box which provided a stable humidity of 95% and a temperature of 25°C as well as sufficient illumination for the cuttings. The rooting rate of the cuttings which were collected from the 1-year old Masson pine tree was 31%, while the rooting rate of the same cuttings managed with the full-light automatic spraying method was 95%. It indicates that stable temperature, humidity and sufficient light are not sufficient to promote the rooting of Masson pine cuttings and explains the importance of a well-aerated environment for the rooting of Masson pine cuttings.

The rooting rate and root quality of stem cuttings dropped significantly when needles were removed from the lower part of cuttings and the cuttings were trimmed diagonally. The removal of needles reduced the photosynthetic units of cuttings, which weakened their carbon assimilation capacity. Studies show that the development of adventitious roots is positively correlated with the photosynthetic rate and carbohydrate content of cuttings [47, 48]. In hazelnut, reducing the net CO_2_ assimilation of leafy cuttings by inducing shade decreased their non-structural carbohydrate content and rooting rate [49].

Stem cuttings collected in May showed significantly higher rooting rate and better root quality than those collected in June and July, while cuttings collected in June showed better performance than those collected in July. He *et al*. [25] reported that the rooting rate of Masson pine cuttings is affected by the degree of lignification, and semi-hardwood cuttings exhibit a higher rooting rate than hardwood cuttings. In Hefei, stem lignification begins in April and gradually progresses in the following months. Our results indicate that the rooting capacity of stem cuttings decrease with the increase in lignification. In many plant species, juvenile cuttings show better adventitious rooting than mature cuttings [50]. This may be due to the presence of higher levels of growth-promoting substances, such as plant hormones, or the absence of inhibitors in juvenile cuttings compared with mature cuttings. A study on *Eucalyptus globulus* points out the loss of rooting capacity of mature plants is related to the decrease of the expression of auxin synthesis genes and receptor genes and increase of expression of auxin response repression-associated genes and rhizogenesis inhibitor-related genes in plants with lower rooting competence [51]. In Loblolly pine an auxin-inducible gene is found differently expressed in mature and juvenile-phase shoots [52]. The effect of lignification on the rooting ability of Masson pine may also be related to changes in the expression patterns of the rooting-related genes.

In the present study, we demonstrated the effects of five factors on the rooting of PWD resistant Masson pine clones and found the optimal management method, substrate, cutting material, cutting treatment, and collection time. The results provide a strong foundation for the development of a cheap, reliable, and simple cutting methodology for propagating PWN resistant clones. The young resistant clone plants produced can be used for restoring the pine forests killed by PWN and afforesting areas threatened by PWD which will improve the resistance of pine forests to PWD and block its further spread.

Masson pine is difficult to root because it contains substances that inhibit rooting, and its rooting rate decreases significantly as the inhibitor content increases with age. The rooting rate of 9-year-old Masson pine cuttings managed by the fully enclosed intermittent spray method was 31% and increased to 57.90% when repeated cuttings with the seedling obtained [36]. The rooting rate of 8-year-old Masson pine cuttings managed with full-light automatic spray management and shaded at the first two weeks was 22% with only one adventitious root and 3 lateral roots, and a total root length of 2.224cm [35]. In the present study, the rooting rate of 10-year-old Masson pine cuttings reached 75.17%, which was 17.27% higher than that of rejuvenated 9-year-old Masson pine cuttings and the number of adventitious root and lateral root number, and total root length of the 10-year-old Masson pine cuttings were much higher than that of the 8-year-old Masson pine cuttings. The cutting method used showed a great effect in promoting the rooting of mature Masson pine cuttings. It provides a technical reference for the cutting of other pine tree species that are difficult to root or lose the rooting ability for aged [52]. Besides, the cutting methodology summarised is simple and cheap. In spring and summer, it needs no glasshouse and temperature, humidity and light control system [53], which make it a good choice for vegetative reproduction of pine tree.

In this article, the affection of management methods, substrates, cutting materials, cutting treatments, and collection times on rooting of PWD resistant clones were individually analyzed and found the best treatment for each factor. However, the interaction between the various factors has not been studied. Combinations of these different factors could be more beneficial to the rooting of PWD resistant clone cuttings. To study the interaction and combination effects of factors that affect the rooting of Masson pine through orthogonal experiments is our next research plan.

## Conclusions

Overall, our results showed that PWD resistant Masson pine clones could be vegetatively propagated in Hefei (Anhui) using cuttings. The cuttings should ideally be collected in May, with a horizontal cut, and the needles should be retained to maximize their rooting potential. Additionally, using perlite (substrate) and the full-light automatic spray management method would further enhance the rooting rate and other root related traits of seedlings.

## Supporting information

S1 Table Effect of management methods on the rooting rate of PWD resistant Masson pine stem cuttings. (DOC)

S2 Table Effect of the cutting material on rooting rate and other root related traits of PWD resistant stem cuttings. (DOC)

S3 Table Effect of substrate on the rooting rate and other root related traits of PWD resistant stem cuttings. (DOC)

S4 Table Effect of cutting treatments on the rooting rate and other root related traits of PWD resistant stem cuttings. (DOC)

S5 Table Effect of collection time on the rooting rate and other root related traits of PWD resistant stem cuttings. (DOC)
