# Supplementary material for: Optimization of factors affecting the rooting of pine wilt disease resistant Masson pine (Pinus massoniana) stem cuttings
Source: PLoS One. 2021 Sep 10;16(9):e0251937. doi: 10.1371/journal.pone.0251937 (PMC8432847; doi:10.1371/journal.pone.0251937)
Supplement: S1 Table — (DOC) [file pone.0251937.s001.doc]

**S1 Table. Effect of management methods on the rooting rate of PWD resistant Masson pine stem cuttings.**

| **Management method** | **Repeats** | **Rooting rate** ^a^ | | | |
| --- | --- | --- | --- | --- | --- |
|  |  | **Sep-17** | **Dec-17** | **May-18** | **Jul-18** |
| Fully enclosed internal circulation | 1 | 0% | 0% | 0% | 0% |
|  | 2 | 0% | 0% | 0% | 0% |
|  | 3 | 0% | 0% | 0% | 0% |
|  | 4 | 0% | 0% | 0% | 0% |
|  | 5 | 0% | 0% | 0% | 0% |
|  | 6 | 0% | 0% | 0% | 0% |
| Fully enclosed intermittent spray | 1 | 0% | 0% | 0% | 0% |
|  | 2 | 0% | 0% | 0% | 0% |
|  | 3 | 0% | 0% | 0% | 0% |
|  | 4 | 0% | 0% | 0% | 0% |
|  | 5 | 0% | 0% | 0% | 0% |
|  | 6 | 0% | 0% | 0% | 0% |
| Full-light automatic spray | 1 | na | na | 54% | 9% |
|  | 2 | na | na | 69% | 21% |
|  | 3 | na | na | 65% | 23% |
|  | 4 | na | na | 80% | 19% |
|  | 5 | na | na | 64% | 24% |
|  | 6 | na | na | 31% | 12% |

^a^na, not applicable.
