# Supplementary material for: Optimization of factors affecting the rooting of pine wilt disease resistant Masson pine (Pinus massoniana) stem cuttings
Source: PLoS One. 2021 Sep 10;16(9):e0251937. doi: 10.1371/journal.pone.0251937 (PMC8432847; doi:10.1371/journal.pone.0251937)
Supplement: S2 Table — (DOC) [file pone.0251937.s002.doc]

**S2 Table. Effect of the cutting material on rooting rate and other root related traits of PWD resistant stem cuttings.**

| **Cutting material** | **repeat** | **number of cuttings** | **Rooting rate** | **Rooting effect index** | **plant sample** | **Root measurements** | | | | |
| --- | --- | --- | --- | --- | --- | --- | --- | --- | --- | --- |
|  |  |  |  |  |  | **No. of adv. roots** | **Adv. root diameter (mm)** | **Longest adv. root length(cm)** | **Total root length (cm)** | **No. of lateral roots** |
|  |  |  |  |  |  |  |  |  |  |  |
| Stem | 1 | 22 | 9.09% | 0.10 | 1-1 | 2.00 | 2.58 | 17.80 | 27.00 | 4.00 |
|  | 2 | 24 | 20.83% | 1.04 | 1-2 | 3.00 | 1.51 | 11.20 | 23.30 | 2.00 |
|  | 3 | 22 | 22.73% | 0.55 | 2-1 | 9.00 | 1.90 | 17.40 | 87.50 | 19.00 |
|  | 4 | 31 | 19.35% | 0.60 | 2-2 | 6.00 | 2.34 | 21.60 | 152.40 | 87.00 |
|  | 5 | 37 | 24.32% | 0.28 | 3-1 | 2.00 | 1.48 | 12.80 | 28.00 | 18.00 |
|  | 6 | 26 | 11.54% | 0.51 | 3-2 | 7.00 | 1.12 | 17.40 | 79.40 | 44.00 |
|  |  |  |  |  | 4-1 | 3.00 | 1.62 | 10.30 | 55.80 | 39.00 |
|  |  |  |  |  | 4-2 | 1.00 | 2.67 | 18.20 | 136.90 | 18.00 |
|  |  |  |  |  | 5-1 | 1.00 | 0.88 | 3.60 | 7.40 | 9.00 |
|  |  |  |  |  | 5-2 | 2.00 | 1.55 | 6.80 | 36.10 | 21.00 |
|  |  |  |  |  | 5-3 | 5.00 | 1.44 | 6.80 | 44.30 | 54.00 |
|  |  |  |  |  | 5-4 | 3.00 | 1.31 | 13.50 | 85.50 | 67.00 |
|  |  |  |  |  | 6-1 | 1.00 | 2.81 | 11.20 | 18.70 | 6.00 |
|  |  |  |  |  | 6-2 | 2.00 | 2.17 | 8.50 | 22.40 | 14.00 |
|  |  |  |  |  | 6-3 | 3.00 | 2.48 | 16.20 | 303.60 | 82.00 |
| Needle brunch | 1 | 19 | 5.26% | 0.01 | 1-1 | 2.00 | 0.60 | 2.30 | 4.10 | 3.00 |
|  | 2 | 18 | 5.56% | 0.01 | 1-2 | 1.00 | 0.73 | 0.70 | 0.70 | 0.00 |
|  | 3 | 19 | 5.26% | 0.01 | 2-1 | 2.00 | 0.72 | 1.30 | 2.60 | 0.00 |
|  | 4 | 13 | 0.00% | 0.00 | 3-1 | 2.00 | 0.39 | 3.10 | 3.80 | 3.00 |
|  | 5 | 10 | 0.00% | 0.00 |  |  |  |  |  |  |
|  | 6 | 12 | 0.00% | 0.00 |  |  |  |  |  |  |
