# Supplementary material for: Optimization of factors affecting the rooting of pine wilt disease resistant Masson pine (Pinus massoniana) stem cuttings
Source: PLoS One. 2021 Sep 10;16(9):e0251937. doi: 10.1371/journal.pone.0251937 (PMC8432847; doi:10.1371/journal.pone.0251937)
Supplement: S3 Table — (DOC) [file pone.0251937.s003.doc]

**S3 Table. Effect of substrate on the rooting rate and other root related traits of PWD resistant stem cuttings.**

| **Substrate** | **repeat** | **number of cuttings** | **Rooting rate** | **Rooting effect index** | **plant sample** | **Root measurements** | | | | |
| --- | --- | --- | --- | --- | --- | --- | --- | --- | --- | --- |
|  |  |  |  |  |  | **No.** of adv. roots | **Adv. root diameter (**mm) | **Longest adv. root length(cm)** | **T**otal root length (cm) | **No.** of lateral roots |
|  |  |  |  |  |  |  |  |  |  |  |
| Mixed substrate | 1 | 11 | 9.09% | 0.00 | 1-1 | 0 | 0.00 | 0.00 | 0.00 | 0.00 |
|  | 2 | 12 | 58.33% | 2.25 | 2-1 | 4 | 0.85 | 9.30 | 46.30 | 48.00 |
|  | 3 | 11 | 45.45% | 2.19 | 3-1 | 3 | 1.46 | 6.30 | 53.10 | 20.00 |
|  | 4 | 13 | 38.46% | 4.21 | 4-1 | 7 | 0.73 | 14.10 | 142.30 | 83.00 |
|  | 5 | 11 | 9.09% | 0.22 | 5-1 | 2 | 1.33 | 6.90 | 27.00 | 14.00 |
|  | 6 | 13 | 30.77% | 1.22 | 6-1 | 4 | 1.05 | 8.90 | 51.40 | 28.00 |
| Sand | 1 | 20 | 80.00% | 12.10 | 1-1 | 27 | 0.76 | 25.70 | 428.20 | 418.00 |
|  | 2 | 19 | 94.74% | 3.34 | 1-2 | 12 | 0.61 | 9.40 | 177.00 | 237.00 |
|  | 3 | 20 | 55.00% | 1.02 | 2-1 | 3 | 1.39 | 7.70 | 20.10 | 16.00 |
|  | 4 | 14 | 64.29% | 3.92 | 2-2 | 6 | 0.87 | 20.20 | 113.70 | 172.00 |
|  | 5 | 17 | 88.24% | 7.21 | 3-1 | 3 | 1.29 | 9.80 | 39.20 | 91.00 |
|  | 6 | 16 | 68.75% | 3.58 | 3-2 | 5 | 1.01 | 11.50 | 34.80 | 35.00 |
|  |  |  |  |  | 4-1 | 10 | 1.15 | 8.60 | 85.40 | 66.00 |
|  |  |  |  |  | 5-1 | 9 | 0.95 | 7.00 | 51.50 | 109.00 |
|  |  |  |  |  | 5-2 | 8 | 0.79 | 26.50 | 226.50 | 254.00 |
|  |  |  |  |  | 6-1 | 4 | 1.27 | 8.40 | 60.60 | 59.00 |
|  |  |  |  |  | 6-2 | 7 | 0.96 | 10.50 | 106.10 | 137.00 |
| Perlite | 1 | 13 | 53.85% | 5.79 | 1-1 | 5 | 1.29 | 18.10 | 139.90 | 103.00 |
|  | 2 | 16 | 68.75% | 15.71 | 2-1 | 6 | 1.05 | 17.20 | 117.10 | 214.00 |
|  | 3 | 20 | 65.00% | 16.21 | 2-2 | 8 | 1.36 | 36.00 | 613.90 | 415.00 |
|  | 4 | 15 | 80.00% | 9.81 | 3-1 | 9 | 1.01 | 35.00 | 423.00 | 337.00 |
|  | 5 | 14 | 64.29% | 10.07 | 3-2 | 20 | 0.74 | 32.40 | 574.60 | 564.00 |
|  | 6 | 16 | 31.25% | 1.96 | 4-1 | 14 | 0.56 | 13.50 | 122.30 | 55.00 |
|  |  |  |  |  | 4-2 | 12 | 0.98 | 17.90 | 245.40 | 97.00 |
|  |  |  |  |  | 5-1 | 6 | 1.19 | 22.10 | 219.40 | 145.00 |
|  |  |  |  |  | 6-1 | 6 | 0.94 | 21.90 | 144.80 | 80.00 |
|  |  |  |  |  | 6-2 | 3 | 0.75 | 15.10 | 56.10 | 35.00 |
| Nutrient soil | 1 | 16 | 50.00% | 4.95 | 1-1 | 4 | 1.08 | 11.30 | 144.30 | 103.00 |
|  | 2 | 16 | 18.75% | 0.82 | 1-2 | 4 | 0.72 | 13.40 | 172.30 | 90.00 |
|  | 3 | 20 | 45.00% | 2.20 | 2-1 | 6 | 0.65 | 5.10 | 47.50 | 134.00 |
|  | 4 | 20 | 55.00% | 1.39 | 2-2 | 7 | 1.00 | 11.20 | 91.60 | 91.00 |
|  | 5 | 17 | 64.71% | 4.02 | 3-1 | 3 | 0.79 | 23.00 | 67.70 | 32.00 |
|  | 6 | 17 | 41.18% | 1.48 | 3-2 | 6 | 0.50 | 23.30 | 127.70 | 118.00 |
|  |  |  |  |  | 4-1 | 1 | 1.07 | 2.30 | 4.70 | 1.00 |
|  |  |  |  |  | 4-2 | 4 | 0.53 | 15.90 | 96.20 | 114.00 |
|  |  |  |  |  | 5-1 | 4 | 1.27 | 12.10 | 107.00 | 78.00 |
|  |  |  |  |  | 5-2 | 4 | 0.94 | 25.20 | 104.30 | 69.00 |
|  |  |  |  |  | 6-1 | 2 | 0.95 | 3.20 | 50.60 | 27.00 |
|  |  |  |  |  | 6-2 | 3 | 1.12 | 8.70 | 71.60 | 33.00 |
