# Supplementary material for: Optimization of factors affecting the rooting of pine wilt disease resistant Masson pine (Pinus massoniana) stem cuttings
Source: PLoS One. 2021 Sep 10;16(9):e0251937. doi: 10.1371/journal.pone.0251937 (PMC8432847; doi:10.1371/journal.pone.0251937)
Supplement: S4 Table — (DOC) [file pone.0251937.s004.doc]

**S4 Table. Effect of cutting treatments on the rooting rate and other root related traits of PWD resistant stem cuttings.**

| **Cutting treatment** | **repeat** | **number of cuttings** | **Rooting rate** | **Rooting effect index** | **plant samples** | **Root measurements** | | | | |
| --- | --- | --- | --- | --- | --- | --- | --- | --- | --- | --- |
|  |  |  |  |  |  | **No.** of adv. roots | **Adv. root diameter (**mm) | **Longest adv. root length(cm)** | **T**otal root length (cm) | **No.** of lateral roots |
|  |  |  |  |  |  |  |  |  |  |  |
| Partially cleaned/diagonal trim | 1 | 14 | 7.14% | 0.24 | 1-1 | 4.00 | 1.49 | 9.60 | 47.40 | 38.00 |
|  | 2 | 15 | 13.33% | 0.17 | 2-1 | 1.00 | 1.50 | 3.40 | 6.40 | 10.00 |
|  | 3 | 18 | 11.11% | 0.11 | 2-2 | 4.00 | 0.52 | 6.90 | 31.10 | 46.00 |
|  | 4 | 16 | 6.25% | 0.16 | 3-1 | 2.00 | 0.86 | 8.50 | 20.70 | 19.00 |
|  | 5 | 12 | 0.00% | 0.00 | 3-2 | 2.00 | 0.86 | 7.20 | 15.10 | 10.00 |
|  | 6 | 12 | 16.67% | 1.15 | 4-1 | 1.00 | 1.24 | 16.20 | 41.50 | 30.00 |
|  |  |  |  |  | 5-1 | 5.00 | 0.87 | 13.90 | 32.80 | 16.00 |
|  |  |  |  |  | 6-1 | 7.00 | 0.97 | 13.20 | 82.80 | 70.00 |
| Intact/horizontal trim | 1 | 12 | 33.33% | 4.08 | 1-1 | 2.00 | 2.37 | 10.10 | 147.00 | 155.00 |
|  | 2 | 15 | 33.33% | 2.35 | 2-1 | 2.00 | 2.28 | 25.90 | 111.50 | 103.00 |
|  | 3 | 16 | 18.75% | 1.05 | 2-2 | 2.00 | 1.51 | 10.80 | 100.00 | 133.00 |
|  | 4 | 14 | 50.00% | 5.39 | 3-1 | 1.00 | 1.83 | 20.20 | 47.00 | 21.00 |
|  | 5 | 8 | 62.50% | 18.12 | 3-2 | 5.00 | 1.47 | 21.80 | 132.10 | 100.00 |
|  | 6 | 8 | 0.00% | 0.00 | 4-1 | 1.00 | 1.79 | 29.20 | 150.80 | 168.00 |
|  |  |  |  |  | 5-1 | 2.00 | 2.70 | 21.90 | 231.90 | 194.00 |
